# Studying the Possibilities of Using 2-Halogen-Substituted Acetamides As Acyl Donors in Penicillin Acylase-Catalyzed Reactions

**DOI:** 10.32607/20758251-2019-11-2-77-81

**Published:** 2019

**Authors:** N. V. Panin, M. V. Nikulin, E. S. Tiurin, V. V. Drobot, I. A. Morozova, V.K. Švedas

**Affiliations:** Lomonosov Moscow State University, Belozersky Institute of Physicochemical Biology, Lenin Hills 1 , bldg. 40, Moscow, 119991, Russia; Lomonosov Moscow State University, Department of Chemistry, Lenin Hills 1, bldg. 3, Moscow, 119991 , Russia

**Keywords:** Penicillin acylases, substrate specificity, 2-haloacetamides, inactivation during the reaction

## Abstract

The possibility of using amides of halogen-substituted acetic acids as acyl donors in penicillin acylase-catalyzed reactions has been investigated, and the ability of this group of compounds to inactivate enzymes in the course of the catalytic conversion has been established. The strongest inactivating effect was demonstrated by iodoacetamide and bromoacetamide. However, the negative contribution of this side activity can be minimized by decreasing the temperature, when the rate of acyl donor conversion by penicillin acylases is still high enough, but the impact of enzyme inactivation becomes less significant. The catalytic activity of penicillin acylase from *Alcaligenes faecalis *in the conversion of 2-haloacetamides was significantly (5–8 times) higher than that of penicillin acylase from *Escherichia coli*.

## INTRODUCTION

**Fig. 1:**
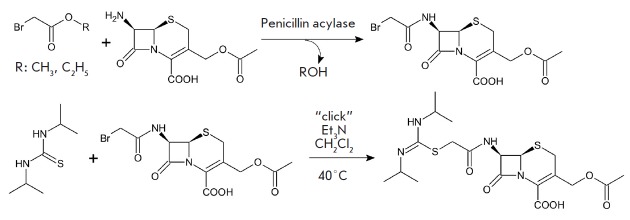
Synthesis of antibiotic cefathiamidine from the activated N-bromoacetyl derivative of 7-aminocephalosporanic acid

Beta-lactam antibiotics are the most widely used antibacterial drugs of high clinical efficacy and low toxicity. The wide availability of the representatives of this antibiotic class is of great practical value, mainly due to the wide application of biocatalytic technologies in their production. However, the emerging resistance of pathogens to antibiotics limits the period of potency of the developed drugs and makes necessary a search for new derivatives. One of the key routes to creating more efficient semisynthetic analogues is to introduce novel N-acyl substituents covalently bound to the beta-lactam nucleus. The success of this process is directly related to the availability, efficiency, and ease of insertion of such N-acyl groups into the structure of the target compound. The ability of penicillin acylases to catalyze the effective acyl transfer (primarily of D-phenylglycine and *p*-hydroxy-D-phenylglycine residues from their amides and esters to the penicillin and cephalosporin nuclei) has played an important role in addressing the problem of antibiotic resistance. A detailed study of the complicated kinetics of enzymatic acyl transfer to external nucleophiles in aqueous media has revealed the major factors defining the efficiency of the process [1-3]. It allowed researchers to develop methods for the biocatalytic synthesis of ampicillin, amoxicillin, cephalexin, cefaclor, cefonicid, and cefprozil in an aqueous medium without using environmentally harmful organic solvents [4-11]. Further development of biocatalytic methods largely depends on the substrate specificity of the enzymes that are capable of catalyzing the transfer of other acyl groups to the nuclei of beta-lactam compounds. Thus, a combination of biocatalysis and click chemistry seems to be a promising direction, where enzyme-catalyzed synthesis of a beta-lactam compound with an N-acyl substituent containing an activated group is performed as the first step. This beta-lactam compound can then be used as a click-starting material to produce a variety of new derivatives. Synthesis of cefathiamidine, a popular antibiotic on the Chinese pharmaceutical market, can be an example to illustrate this approach. In this case, the activated N-acyl group is a residue of bromoacetic acid. Subsequent reaction of this group with *N,N’*-diisopropylthiourea yields the desired antibiotic (*Fig. 1*) [12, 13].

A sufficiently versatile method for obtaining a wide range of potential antibacterial drugs can be developed by varying the structure of the residue enzymatically inserted into the antibiotic core, the chemical nature of the activated groups, and the structure of the compound used at the click chemistry step.

One can expect the development of such an approach to be complicated by several factors: chemical modification (or even inactivation) of the enzyme due to interaction with activated groups of the initial substrates or final products, spontaneous destruction of the activated groups under the conditions of the biocatalytic reaction, and the necessity to search for suitable enzymes with the requested catalytic activity toward synthetic nonnatural substrates.

The goal of this work was to investigate the possibilities of using halogen-substituted acetic acid derivatives as potential acyl donors in the reactions catalyzed by penicillin acylases from *Escherichia coli *and *Alcaligenes faecalis*, as well as to study the dependence of their reactivity as substrates and ability to inactivate the enzymes on the nature of the activating group.

## EXPERIMENTAL

**Determination of enzyme activity with respect to 2-haloacetamides**

A typical experiment was carried out as follows: 2-haloacetamide (200 μmol) was dissolved in 0.05 M phosphate buffer, thermostated at the desired temperature, and pH was adjusted to 7.5. The total volume of the reaction mixture was 800 μl; the substrate concentration was 0.25 M. The reaction was then started by adding an aliquot of the concentrated enzyme solution so that the concentration of active sites of penicillin acylase in the reaction mixture was 25 μM. The reaction was carried out in a thermostated cell; temperature and pH were maintained constant. Aliquots of 20 μl were sampled at regular intervals and mixed with 980 μl of a stock solution, which was a mixture of acetonitrile and distilled water at a 2:1 ratio (v/v). The samples were centrifuged for 5 min at 13,000 rpm in order to remove the precipitated protein and subjected to reverse phase HPLC analysis. Analysis conditions were as follows: flow rate, 0.7 ml/min; acetonitrile/ water (25% acetonitrile v/v, 0.005 M phosphate buffer pH 3) used as an eluent; detection at 210 nm; Kromasil Eternity 5-C18 4.6×250 mm column; and sample volume, 20 μl. Chromatographic resolution RS of the corresponding amide and acid exceeded 1.5 in all cases. The retention times of the components were as follows: chloroacetamide, 4.16 min; chloroacetic acid, 4.34 min; bromoacetamide, 4.27 min; bromoacetic acid, 4.73 min; iodoacetamide, 4.31 min; and iodoacetic acid, 5.92 min.

**Studying the dependence between the conversion rate of the chromogenic substrate and the 2-chloroacetamide concentration**

The inhibitory influence of chloroacetamide on the catalytic activity of penicillin acylase was studied using a CLARIOstar high-performance microplate reader (BMG LABTECH). A typical experiment was conducted using the following procedure: aliquots of a 1 mM chromogenic substrate, NIPAB solution, were added to the cells of a 96-well plate in order to create seven vertical columns with the NIPAB concentrations ranging from 0.01 to 0.3 mM, whereas the substrate concentrations in the horizontal rows were equal. Aliquots of a 700 mM inhibitor solution were then added to the cells along the horizontal rows in order to create eight rows of various inhibitor concentrations ranging from 0 to 400 mM, whereas the inhibitor concentrations in the columns were equal. The volume of the reaction mixture in each cell was adjusted to 216 μl by adding the required volume of 0.1 M potassium phosphate buffer (pH 7.5). Solutions of all the reagents were prepared in the same buffer. Reactions were started by adding 20 μl of the stock enzyme solution using a multichannel dispenser; the concentration of active sites of penicillin acylase in each cell was 10 nM. The temperature was maintained at 25°C. The enzyme activity was monitored as an accumulation of chromophore, *p*-nitro-*m*-carboxyaniline, at 400 nm in the Absorbance/PlateMode operating mode of increased accuracy (number of flashes per cell, 30; cycle time, 6 s; number of cycles, 74) with periodic mixing (500 rpm). To avoid random errors caused by the formation of local air bubbles, the absorption was measured in the statistical averaging mode (well scan function, spiral averaging). Stream regression data processing was carried out using the MARS Data Analysis software. The initial reaction rates were determined as the average value of the derivative within 10% of the NIPAB conversion. To determine the inhibition constant, the experimental data were analyzed in Dixon coordinates.

**Studying penicillin acylase inactivation by 2-haloacetamides**

The inactivation kinetics of penicillin acylase was studied using a CLARIOstar high-performance microplate reader (BMG LABTECH). A typical experiment was as follows: aliquots of a 1 M 2-haloacetamide solution were added to the cells of a 96-well plate in order to create eight rows with different 2-haloacetamide concentrations ranging from 0 to 470 mM, whereas the concentrations in all 12 columns were equal. The left half of the plate was used to study enzyme inactivation by 2-haloacetamide alone, while the right half was used to study the influence of phenylacetic acid (PAA), a highly specific competitive inhibitor of penicillin acylase, on this inactivation. The PAA concentration in all cells of the right half of the plate was 0.1 mM. The volume of the reaction mixture in each cell was adjusted to 196 μl by adding the required volume of 0.1 M potassium phosphate buffer (pH 7.5). Thus, six columns with an identical composition of reagents were created on the left part (without PAA) and on the right part (with PAA). The inactivation reaction was started simultaneously in the first and the seventh column by adding 20 μl of the enzyme stock solution, so that the concentration of the active sites of penicillin acylase in each cell was 10 nM. The aliquots of the enzyme stock solution were sequentially added to each following column using a multichannel dispenser after every 10 minutes of incubation. The temperature was maintained at 25°C. In order to determine the residual enzyme activity 50 min after the first incubation had been started, 24 μl of a 1 mM NIPAB solution was simultaneously added to all the cells. Thus, the concentration of the chromogenic substrate in each cell was 0.1 mM. The time of incubation of the enzyme in the first and seventh columns was 50 min; in the sixth and twelfth columns, 1 min. The residual activity of penicillin acylase was monitored as described above. Enzyme inactivation proceeded according to the first-order reaction kinetics; the corresponding inactivation constant was determined by analyzing the obtained experimental data.

The kinetics of inactivation of penicillin acylase by 2-haloacetamides was also studied using another technique. The enzyme solution (concentration of the active sites, 3 nM) was incubated with 100 mM 2-haloacetamide in 10 mM potassium phosphate buffer (pH 7.5) containing 0.1 M KCl at different temperatures (4, 15 and 25°C). Aliquots of the reaction mixture were sampled at regular intervals and added to a thermostated cuvette (25°C) containing a 0.1 mM NIPAB solution in 10 mM potassium phosphate buffer (pH 7.5) and 0.1 M KCl. The residual activity of the enzyme was monitored using a Shimadzu UV 1800 spectrophotometer at 400 nm.

## RESULTS AND DISCUSSION

**Selection of halogen-substituted acetic acid derivatives as potential substrates for research**

The properties of a chemical compound, such as stability, solubility and usability, should be considered when choosing a substrate for an enzymatic reaction. It is especially important to bear in mind the preparative use of acyl donors. When selecting halogen-substituted acetamides for their study as potential substrates of penicillin acylases, the crucial factors were as follows: higher solubility and stability of amides compared to those of esters and, most importantly, the lachrymatory properties of the corresponding esters. Thus, vapors of ethyl bromoacetate are extremely irritating to the eye mucosa. This substance should be stored in a hermetically sealed vessel and operated with in an open vessel only in a well-functioning fume hood [14].

**Studying the ability of 2-haloacetamides to bind in the active site of penicillin acylases**

**Fig. 2:**
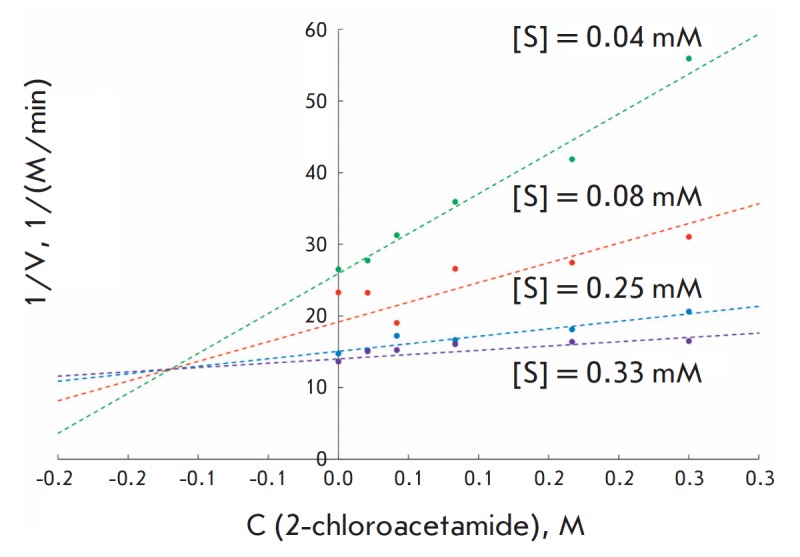
Dependence between the initial rates of NIPAB hydrolysis catalyzed by penicillin acylase from *Escherichia coli *and concentration of the chromogenic substrate in the presence of various concentrations of 2-chloroacetamide. The experimental data are presented in Dixon coordinates. Experimental conditions: 25°C, 0.1 M potassium phosphate buffer (pH 7.5)

Potential substrates were studied for their ability to bind in the active site of penicillin acylase and thus inhibit its activity toward a chromogenic substrate. A typical example of the influence of 2-chloroacetamide on the catalytic activity of penicillin acylase from *Escherichia coli *is shown in *Fig. 2*. An analysis of the dependence between the conversion rate of NIPAB and the 2-chloroacetamide concentration showed that this compound is a competitive inhibitor with an inhibition constant of 0.12 ± 0.02 M (*Fig. 2*). It was observed that 2-haloacetamides possess lower affinity to the active site of penicillin acylases (by about four orders of magnitude) compared to that of specific substrates of this enzyme. Nevertheless, due to the high solubility of

**Table 1:** The rate constants of inactivation of penicillin acylase from Escherichia coli upon enzyme interaction with 2-haloacetamides

Substrate	kin∙10^4^, min^-1^
Chloroacetamide	1.1 ± 0.1
Bromoacetamide	47 ± 2
Iodoacetamide	364 ± 34

^*^Experimental conditions: 25°C, 0.1 M potassium phosphate buffer (pH 7.5), 0.1 M 2-haloacetamide

It was found that enzymatic hydrolysis of 2-haloacetamides is accompanied by inactivation of penicillin acylases. Inactivation especially prevailed in the case of hydrolysis of 2-iodoacetamide and 2-bromoacetamide. Inactivation of both enzymes was caused by binding of haloacetamides in the active site of penicillin acylases and was associated with their catalytic activity, since the addition of phenylacetic acid (a known competitive penicillin acylase inhibitor that binds in the active site) suppressed inactivation. Similar inactivation of penicillin acylase from *E.coli *in the course of a catalytic reaction was previously observed upon preparative enzymatic synthesis of D-phenylglycyl peptides [15]. Loss of enzyme activity upon conversion of the amides of halogen-substituted acetic acids proceeded according to first-order reaction kinetics; the corresponding inactivation rate constants are presented in *Table 1*.

**Fig. 3:**
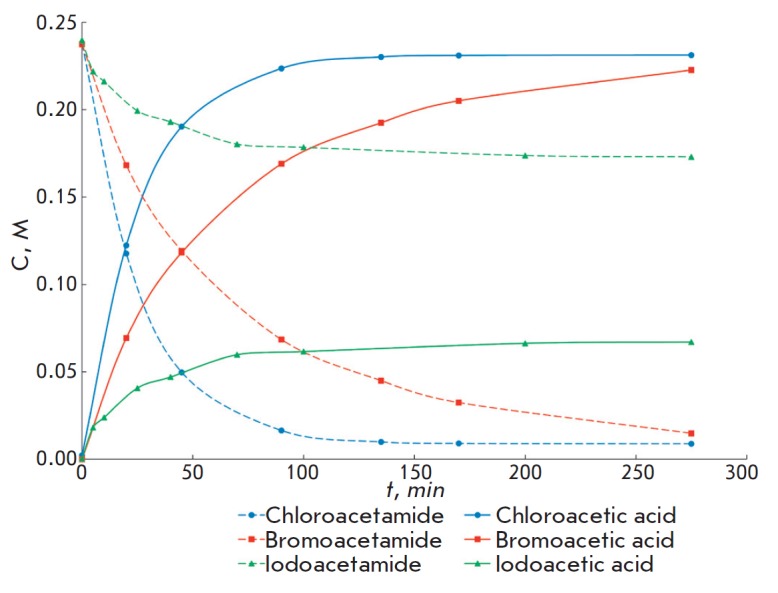
Accumulation of the reaction products (solid curves) and consumption of the initial substrates (dashed curves) in the course of hydrolysis of 2-haloacetamides catalyzed by penicillin acylase from *Alcaligenes faecalis*. The reaction with chloroacetamide was carried out at 25°C; with bromo- and iodoacetamide, at 4°C. The experimental conditions are shown in the caption to *Table 2*

Inactivation of penicillin acylase from *E.coli *by 2-iodoacetamide at an optimal pH of the enzyme and 25°C proceeded so rapidly that the substrate was hydrolyzed only to several percents. Enzyme inactivation in the presence of 2-bromoacetamide was slower, but it also prevented the use of this acyl donor in preparative synthesis under these conditions. We succeeded in slowing down the enzyme inactivation rate and suppressing the negative impact of this process on the catalytic conversion of acyl donors by reducing the temperature (*Fig. 3*). Thus, the loss of enzyme activity was decreased by more than an order of magnitude at 4°C upon hydrolysis 2-bromoacetamide, which makes it possible to use this compound as an acyl donor in reactions catalyzed by penicillin acylases under these conditions. It should also be noted that penicillin acylase from *Alcaligenes faecalis *was more active with respect to this group of substrates compared to the enzyme from *Escherichia coli* (see *Table 2*).

**Table 2:** Specificity of penicillin acylases in 2-haloacetamides hydrolysis

Substrate \ Enzyme	Penicillin acylase from E. *coli*	Penicillin acylase from A. *faecalis*
	V, μM/s	
Iodoacetamide (4°C)	9.3 ± 1.3	45 ± 2
Bromoacetamide (4°C)	10.0 ± 1.3	58 ± 3
Chloroacetamide (25°C)	7.5 ± 1	63 ± 5

^*^Experimental conditions: 0.05 M potassium phosphate buffer (pH 7.5); the experiment with chloroacetamide was carried out at 25°C; with bromo- and iodoacetamide, at 4°C; the concentration of the active sites of penicillin acylase was 25 μM.

## CONCLUSIONS

The ability of 2-halogen-substituted acetamides to inactivate penicillin acylases in the course of their conversion has been established by studying the possibility of using this group of compounds as acyl donors in biocatalytic acyl transfer reactions. The most efficient inactivation was observed upon conversion of iodoacetamide and bromoacetamide. However, the negative impact of this side activity can be minimized by lowering the temperature, when the catalytic activity of penicillin acylases with these acyl donors remains rather high and the role of enzyme inactivation in the overall process becomes insignificant.

## Abbreviations

HPLC: high-performance liquid chromatography
NIPAB: 2-nitro-5-(phenylacetyl)amino-benzoic acid
PAA: phenylacetic acid
